# Autonomic nervous system imbalance during aging contributes to impair endogenous anti-inflammaging strategies

**DOI:** 10.1007/s11357-023-00947-7

**Published:** 2023-10-11

**Authors:** Sergio Giunta, Shijin Xia, Giuseppe Pelliccioni, Fabiola Olivieri

**Affiliations:** 1 Casa Di Cura Prof. Nobili (Gruppo Garofalo (GHC)), Castiglione Dei Pepoli, Bologna, Italy; 2 Department of Geriatrics, Shanghai Institute of Geriatrics, Huadong Hospital, Fudan University, Shanghai, China; 3 Unit of Neurology, IRCCS INRCA, Ancona, Italy; 4 Department of Clinical and Molecular Sciences, DISCLIMO, Università Politecnica Delle Marche, Via Tronto 10/A, 60126 Ancona, Italy; 5 Clinical Laboratory and Molecular Diagnostic, IRCCS INRCA, Ancona, Italy

**Keywords:** Inflammaging, Anti-inflammaging, Macrophaging, Autonomic imbalance, Increased sympathetic tone, Diminished parasympathetic activity, Cardiovascular-inflammaging, HPA axis, Cholinergic anti-inflammatory pathway (CAP)

## Abstract

Inflammaging refers to the age-related low grade, sterile, chronic, systemic, and long-lasting subclinical, proinflammatory status, currently recognized as the main risk factor for development and progression of the most common age-related diseases (ARDs). Extensive investigations were focused on a plethora of proinflammatory stimuli that can fuel inflammaging, underestimating and partly neglecting important endogenous anti-inflammaging mechanisms that could play a crucial role in such age-related proinflammatory state. Studies on autonomic nervous system (ANS) functions during aging highlighted an imbalance toward an overactive sympathetic nervous system (SNS) tone, promoting proinflammatory conditions, and a diminished parasympathetic nervous system (PNS) activity, playing anti-inflammatory effects mediated by the so called cholinergic anti-inflammatory pathway (CAP). At the molecular level, CAP is characterized by signals communicated via the vagus nerve (with the possible involvement of the splenic nerves) through acetylcholine release to downregulate the inflammatory actions of macrophages, key players of inflammaging. Notably, decreased vagal function and increased burden of activated/senescent macrophages (macrophaging) probably precede the development of several age-related risk factors and diseases, while increased vagal function and reduced macrophaging could be associated with relevant reduction of risk profiles. Hypothalamic–pituitary–adrenal axis (HPA axis) is another pathway related to ANS promoting some anti-inflammatory response mainly through increased cortisol levels. In this perspective review, we highlighted that CAP and HPA, representing broadly “anti-inflammaging” mechanisms, have a reduced efficacy and lose effectiveness in aged people, a phenomenon that could contribute to fuel inflammaging. In this framework, strategies aimed to re-balance PNS/SNS activities could be explored to modulate systemic inflammaging especially at an early subclinical stage, thus increasing the chances to reach the extreme limit of human lifespan in healthy status.

## Introduction

### Sympathetic nervous system (SNS) and parasympathetic nervous system (PNS) changes during aging

The autonomic nervous system (ANS) is one of the main homeostatic regulatory systems in the body. The two interacting arms of ANS, the sympathetic nervous system (SNS) and parasympathetic nervous system (PNS), must be in balance to maintain the general homeostasis of the organism [1]. The SNS and PNS play seemingly opposite but complementary roles, so that they are known to affect the organism’s responses to threat or stressors by modulating “fight or flight” and “rest and digest” responses, respectively, thus orchestrating a fine-tune modulation of the body’s restore after stressful events [1]. However, the influence of SNS and PNS on target organs does not fall on a single continuum, and the relationships between their complex activities vary between individuals and environmental contexts. The homeostatic mechanism ensured by the balance between SNS and PNS is relevant to maintain a healthy condition during the life-course, especially during aging, when the risk to the development and progression of the most common age-related diseases significantly increases [2]. Increased activity of SNS and less PNS tone at rest, referred as ANS dysfunction, is a condition observed in aged subjects, especially in patients affected by the most common age-related diseases [3]. Since SNS and PNS change in an opposite way during aging, it is reasonable that this phenomenon is not a mere gain/loss of function nor an uncontrolled increased/decreased activity, but rather a remodeling process. The interindividual variability in the degree of ANS dysfunction during aging could be due to the interaction between individual genetic make-up and environmental factors [4].

Two primary mechanisms have been hypothesized to explain age-related increase in peripheral SNS activity, such as reduced tonic baroreflex inhibition of “normal” central SNS outflow and a primary increase in central nervous system (CNS)-generated sympathetic nerve discharge [5].

Aging also affects functional and electrophysiologic properties of the PNS, resulting in an opposite trend compared to age-related SNS changes. Decline in nerve conduction velocity, muscle strength, sensory discrimination, and autonomic responses was associated to aging process in animal models and humans [6]. Proinflammatory status induced by reduced vagus nerve output with age was also associated with endothelial dysfunction, a condition that promotes an increased risk of developing the most common age-related diseases [3]. Aging also determines a reduction in terminal and collateral sprouting of regenerated fibers, further limiting the capabilities for target reinnervation and functional restitution, thus complexively slowing PNS’s regeneration speed [7]. Overall, the capabilities for axonal regeneration and reinnervation are maintained throughout life but tend to be delayed and less effective with aging. Notably, chronic inflammation is a factor related to increased failure in nerve regeneration in mammals of advanced age [8].

Over the last few decades, the mechanisms contributing to overactivated SNS and diminished PNS activity during aging were studied mainly in the framework of cardiovascular diseases (CVDs) [9, 10]. Nowadays, chronic SNS overactivity and PNS downflow are considered risk factors for the development of heart failure, as well as of other CVDs and risk factors, such as obesity, hypertension, and metabolic disorders [11].

The ultra-centenarians have significantly higher PNS activity than elderly, and this PNS predominance was proposed as the neuroautonomic feature that could help to protect against CVDs [12]. However, the general question of the specific age-related ANS functional derangement versus compensatory/remodeling mechanisms remains partly unresolved and needs further research [13]. Notably, the SNS and PNS age-related changes are not linearly progressive with age, suggesting complex relationships between different factors. In this framework, a challenging issue now is to identify processes that could link age-related ANS imbalance with an increased risk of developing all the most common age-related diseases. ANS imbalance, characterized by SNS overdrive and diminished PNS activity, has been associated with increased chronic systemic proinflammatory status since 2002 [14]. Inflammaging was firstly described in 2000 as the systemic, chronic, sterile, low-grade proinflammatory conditions that increases with age and that can be measured analyzing the levels of proinflammatory markers in blood and tissues [15].

Here we hypothesized that inflammaging could be one of the mechanisms at the crossroad between ANS imbalance during aging and the increased risk of developing age-related diseases.

### Inflammaging

To disentangle the hypothesized complex relationships between ANS imbalance and inflammaging, it is imperative to disentangle the key molecular pathways that fuel inflammaging.

Overall, inflammaging is associated with an increased circulating level of proinflammatory cytokines, primarily cytokines, such as IL-1, IL-6, TNF-alpha, chemokines, i.e., IL-8, and acute phase proteins, such as C-reactive protein (CRP) and serum amyloid A [16, 17]. The increased circulating levels of proinflammatory mediators is sustained and perpetuated mainly by two mechanisms: (i) the age-related increased burden of activated immune cells, mainly macrophages, and (ii) the age-related increased burden of senescent cells acquiring a senescence associated secretory phenotype (SASP), typically proinflammatory [18, 19].

Both innate and adaptive immune systems can contribute to sustain inflammaging [15, 20, 21]. Innate immune system was the first identified culprit of inflammaging, and macrophages were recognized as the innate immune cells mainly involved in such phenomenon, so that the term “macrophaging” appeared for the first time in the first paper on inflammaging [15].

The recently developed inflammatory aging clock (iAge) based on deep learning showed its ability to predict the aging trajectory and CXCL9, the cytokine produced mainly by macrophages, was identified as the most robust contributor to the iAge, confirming the key role played by macrophages in inflammaging process [22]. Acute inflammatory processes are fundamental host defense mechanisms elicited by infectious and injuries, triggering the healing process. On the contrary, long-term exposure to low-grade chronic proinflammatory state can increase the risk of developing aging-related pathologies, thus negatively impacting on healthy aging [23, 24]. Immune system cells chronically stimulated with infectious, i.e., viruses, or non-infectious agents, i.e., post-translationally modified proteins, and misplaced DNA [cytoplasmic (cy) and cell-free (cf) DNA pools] are believed to be driving forces of inflammaging [25]. Regarding DNA, we recently highlighted as changes in a variety of biochemical characteristics of cy- and cf-DNA, such as the amount of 8-oxo-deoxy-guanosine and 5-methyl-deoxy-cytosine, can potentially affect the capability of these DNA pools to ignite the innate immune system [25].

Senescent cells were also identified as culprits of inflammaging: different types of cells can become senescent, acquiring SASP [26]. Recent evidence suggested that an increased burden of senescent cells, including macrophages in the adipose tissue of obese/diabetic animal models and humans, might underlie a proinflammatory phenotype [27]. From a molecular point of view, aberrant inflammasome activation was observed in senescent cells, associated to defective autophagy and mitophagy, thus perpetuating systemic proinflammatory conditions [28].

The increased burden of senescent cells observed during aging is almost in part due to immunosenescence, entailing changes in innate immune cells activities, i.e., reduced macrophages efferocytosis, and in acquired immune system cells activities, i.e., naïve/memory cell ratio imbalance [29, 30]. The reduction in the number of peripheral blood naïve cells, with a relative increase in the frequency of memory cells, together with reduced macrophages phagocytic activity and increased cytokines release, are considered the hallmarks of immunosenescence [31].

In the last 23 years from the first theoretical paper on inflammaging, a growing body of evidence confirmed the relevance of the interindividual variability in the rate of increase of inflammaging level during aging, as risk factor for morbidity and mortality in elderly individuals [32].

Analyzing inflammaging in the framework of “geroscience theory,” assuming that aging process is the main culprit of the development of the most common age-related diseases, inflammaging can be defined as a “hallmark of aging” and, at the same time, as the most clinically relevant risk factors for the most common age-related diseases [33]. If the molecular and cellular mechanisms that promote inflammaging were extensively investigated in the framework of aging and age-related diseases, few studies were focused on the potential association between ANS imbalance and inflammaging level during aging [34].

It should be remembered that an inflammatory process can worsen not only due to the activation of inflammatory mechanisms but also due to reduced effectiveness of anti-inflammatory systems. At present, several physiological anti-inflammatory processes could be recognized as potential anti-inflammaging strategies [35, 36].

In this review, we focused our discussion on the evidence that link the age-related ANS imbalance with the modulation of inflammaging, focusing our discussion on two ANS related potential anti-inflammaging strategies, such as the cholinergic anti-inflammatory pathway (CAP) and the hypothalamic pituitary adrenal (HPA) axis, which seem to loss efficiency and effectiveness during aging, especially in patients affected by the most common age-related diseases.

## The complex relationship between age-related ANS imbalance and inflammaging/anti-inflammaging

The vagus nerve, which is the longest cranial nerve, serves as the primary nerve of PNS, and it is a mixed nerve, comprising 80% afferent fibers and 20% efferent fibers. Vagus nerve trough afferent fibers senses inflammatory molecules, i.e., cytokines, and transmits signals to the nucleus tractus solitarius (NTS) [37, 38]. Vagal signals to the NTS lead to downregulation of inflammatory molecules through two routes: (1) the recently identified CAP, involving efferent fibers targeting immune system cells, such as lymphocytes and macrophages, in spleen and intestine [39, 40], and (2) the activation of the HPA axis.

The reasoning that immune system could be innervated prompted research on neural circuits that can modulate innate and adaptive immunity. Pathogens-associated or damages-associated molecular patterns (PAMPs and DAMPs) can activate sensory neurons and induce signals that travel from the brainstem to the spleen and other organs. The CAP was described for the first time in 2000 by the Kevin Tracey’s team [14], as an anti-inflammatory reflex in which stimulation of vagal afferents by proinflammatory cytokines resulted in activation of vagal efferents inhibiting the release of proinflammatory cytokines, the so-called vago-vagal reflex or inflammatory reflex. Subsequent studies highlighted the molecular mechanisms of CAP [reviewed in 40]. The efferent vagus nerve transmits a signal to the splenic nerve with subsequent release of noradrenaline (NA) and memory CD4 + T lymphocytes that express the beta-2 adrenaline receptor (β2AR), capture noradrenaline, and release acetylcholine [41]. The CD4 + T lymphocytes express the enzyme responsible for acetylcholine synthesis, so that they can release acetylcholine, that in turn, acts on macrophages expressing the α7nAChR receptor thereby inhibiting the production of proinflammatory cytokines [42]. The suppression of inflammatory cytokine secretion in macrophages by acetylcholine is due to the blockage of the transcription factor nuclear factor-kB (NFκB) translocation from cytosol to the nucleus, to the modulation of the Janus kinase 2/signal transducer and activator of transcription 3 (JAK2/STAT3) pathway and to the inhibition of the inflammasome activation [43–46]. Recently, it was observed that α7nAChRs and α7nAChR-dependent cholinergic signaling are implicated in suppressing the release of high mobility group box 1 (HMGB1), a nonhistone nuclear protein that plays multiple functions including that of endogenous alarmin [47].

Recently, it was confirmed that this reflex suppresses the synthesis and release of TNF-alpha, IL-6, and IL-1β while it enhances the levels of the anti-inflammatory cytokine IL-10 [48]. These data confirmed and reinforced previous evidence highlighted as vagus nerve stimulation was associated with the release of specialized pro-resolving mediators (SPMs), a class of bioactive metabolites, derived from omega-3 fatty acids, that play a central role in the resolution of inflammation [49, 50].

The CAP has been proposed as a key mechanism by which the brain, through the vagus nerve, modulates the immune system in the spleen. However, increasing evidence suggested that the anti-inflammatory effect of the vagus nerve in the intestine is independent from the spleen and T cells [51]. It was demonstrated that the vagus nerve interacts with cholinergic myenteric neurons in close contact with the muscularis macrophages, suggesting that intestinal muscularis resident macrophages expressing α7nAChR are most likely the ultimate target of the gastrointestinal CAP [51]. Notably, macrophages are essential for the maintenance of intestinal homeostasis and appear to be drivers of inflammation in the context of inflammatory bowel disease (IBD) (reviewed in [52]). Numerous studies have established that gut microbiome composition, gut immune function, and gut barrier integrity are involved in the proinflammatory derailment of inflammaging [53]. On the other hand, inflammaging additionally drives the development of aging-like phenotypes, such as microbiota dysbiosis and impaired intestinal barrier, via a broad array of inflammatory mediators.

As mentioned above, vagus nerve can exert a dual anti-inflammatory effect stimulating not only the CAP, targeting spleen and intestine, but also targeting the HPA axis, thus modulating the release of glucocorticoids (GCs) from the adrenal glands. Endogenous GCs are central regulators of immune functions so that their potent immunosuppressive and anti-inflammatory properties have led to their widespread medical use to treat inflammatory disorders [54]. The HPA axis responds to physical and psychological stressors that are known to induce the secretion of catecholamine, epinephrine, and norepinephrine via the SNS and GCs via the adrenal gland. Vagal afferents activated by stressors or by increased amount of circulating proinflammatory cytokines activate neurons which release corticotrophin-releasing factor (CRF) thus inducing the release of adrenocorticotropic hormone (ACTH) by the pituitary to stimulate the release of GCs by the adrenal glands to inhibit peripheral inflammation [55]. HPA axis functions are dysregulated with advancing age, but the modulation of systemic and local GC production and responsiveness of receptors during aging processes is partially unknown. In animal models, the aging phenotype characterized by chronic increased release of proinflammatory cytokine, was associated with a dysfunction of the HPA axis and diminished serum corticosteroid levels. Corticosterone is the major active glucocorticoid in rodents, and its levels are reduced in aged animals in association with an impaired expression and activity of 11β-hydroxysteroid dehydrogenase type 1 (11β-HSD1), an enzyme that determines the extent of cellular glucocorticoid responses, so that macrophages of aged animals are less responsive to cortisol [56]. Although short-term activation of the HPA axis and GC release play anti-inflammatory and adaptive effects, chronic stress-induced activation of the HPA axis can hesitate in different outcomes, such as chronic basal hypersecretion, sensitized stress responses and even adrenal exhaustion associated to reduced release of GC. Notably, in patients with arterial disease, older age was associated with a blunted awakening cortisol response, higher levels of cortisol in the evening, and decreased diurnal variability [57].

It was previously highlighted that in contrast to the HPA axis or the local production of anti-inflammatory cytokines, the CAP plays central role in immune homeostasis, since the modulatory effect of CAP is not only fast, but also integrated with the health status [58].

Overall, SNS overdrive in association with the reduction of PNS activity, characterized by dysfunctional HPA and CAP activities, could be particularly relevant in an early phase of inflammaging, when the pathogenetic mechanisms are active but the clinical manifestation is not already evident.

In the subsequent phases, the elicited multiple central and peripheral inflammaging and anti-inflammaging responses, could draw a complex and variable dynamic scenario characterized by an interindividual variability in the rate of inflammaging increase and aging trajectories.

Different long-term outcomes can be envisaged: (a) a re-established inflammaging/anti-inflammaging balance that increases the chances to reach a healthy longevity; (b) further progression of overt-excessive inflammaging, paving the way to age-related diseases development and progression; and (c) enhancement and reinforcement of anti-inflammaging responses that could induce chronic hypercortisolism, a condition paradoxically associated with increased risk of frailty, cognitive decline, and dementia [59–61].

**Fig. 1:**
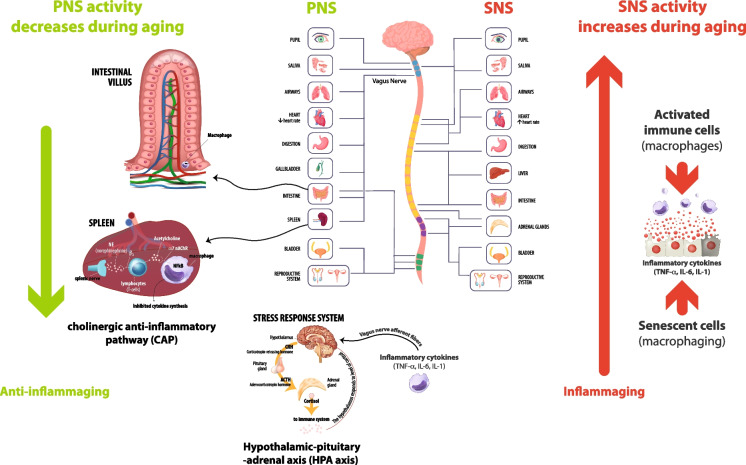
PNS and SNS key activities and relevant age-related changes potentially associated with modulation of inflammaging. PNS and SNS key activities are depicted schematically. HPA and CAP can be stimulated by vagus nerve, acting as anti-inflammatory pathways. In the CAP, the vagus nerve acts in synergy, through the splenic nerve, to inhibit the release of TNFα by macrophages of the spleen. It was recently suggested also a gastrointestinal CAP. Vagus nerve trough afferent fibers senses inflammatory molecules, i.e., cytokines, and transmits signals to the hypothalamus causing the release of CRH inducing then release of ACTH into the systemic circulation, whereby it promotes synthesis and secretion of glucocorticoids at the adrenal cortex. Glucocorticoids are then secreted into the systemic circulation and can access cognate receptors in different organs, including the brain. Reduced HPA and CAP activity during aging can contribute to reduce anti-inflammaging strategies. SNS overflow reported during aging could contribute to fuel inflammaging, acting synergistically with the cellular and molecular culprits of inflammaging, i.e., increased burden of activated immune cells (mainly macrophages) and increased burden of senescent cells (including macrophages). SNS, sympathetic nervous system; PNS, parasympathetic nervous system; HPA axis, hypothalamic pituitary adrenal axis; CAP, cholinergic anti-inflammatory pathway; ACTH, adrenocorticotrophic hormone; CRH, corticotropin releasing hormone; TNFα, tumor necrosis factor-alpha

Figure 1 illustrates the PNS and SNS main activities potentially associated with the modulation of inflammaging and anti-inflammaging.

## ANS imbalance and inflammaging in age-related diseases

Since ANS can regulate inflammation in chronic and acute conditions, autonomic dysfunction may have a pivotal influence on the onset and progression of many age-related diseases, such as autoimmune diseases, cardiovascular diseases (CHD), and ischemic stroke. In these pathologies, commonalities between ANS imbalanced activities and increased inflammaging can be envisaged.

Regarding autoimmune diseases, this is not unexpected, since ANS regulates both innate and adaptive immunity through the sympathetic and parasympathetic branches and its imbalance can determine an altered inflammatory response, as typically observed in rheumatoid arthritis (RA), systemic lupus erythematosus, and systemic sclerosis [62]. These autoimmune diseases show a dysfunction of the ANS that is mutually related to the increase of inflammation. The autonomic imbalance is related to an increased risk of developing cardiovascular disease, which is the major cause of morbidity and mortality in patients affected by autoimmune diseases [63]. More in general, ANS imbalance has been observed in immune-mediated inflammatory diseases (IMIDs), a descriptive term coined for a heterogeneous group of diseases that share common proinflammatory conditions, including not only autoimmune and inflammatory bowel disease, but also obesity, hypertension, chronic pulmonary disease, and coronary heart disease [64]. DNA damage-induced senescence, a mechanism triggering inflammation, could contribute to fuel inflammaging in these apparently unrelated diseases [65].

In the general population, three longitudinal studies such as the ARIC (Atherosclerosis Risk in Communities), the ZUTPHEN, and the FRAMINGHAM studies highlighted the prognostic relevance of unbalanced ANS activity on mortality, especially cardiovascular mortality [9–11]. Interestingly, the Framingham Heart Study identified traditional risk factors for the development of CHD, but many individuals who will develop major adverse cardiovascular events (MACE) were not identified. On the contrary, autonomic dysfunctions have been strongly associated with an elevated risk of cardiac mortality [64]. These results are in line with the observation that chronic inflammation contributes to the residual risk of myocardial infarction and stroke after attaining low levels of low-density lipoprotein cholesterol.

Ischemic stroke is another condition in which imbalance in the ANS and inflammaging levels can be observed. Irrespective of the subtype of the ischemia, post-acute stroke patients are characterized by PNS cardiac deficit [66, 67]. Notably, ischemic stroke is one of the most common causes of death and disability worldwide, and autonomic dysfunction has been observed in 25–76% of patients with acute stroke [68]. Neuroinflammation represents a major pathological event involved in the process of ischemic injury and repair, where microglia, the only macrophage population in the CNS parenchyma, plays key role in neuroinflammation. Emerging evidence indicates that microRNAs (miRNAs) may have regulatory effects on microglia-associated inflammation [69].

Overall, the evidence of an interplay between ANS imbalance and age-related diseases characterized by increased inflammaging levels is multifaceted and further studies on this issue could highlight more commonality among ANS imbalance and inflammaging in additional age-related diseases.

## ANS imbalance and inflammaging in the heart: focus on heart rate variability (HRV)

The heart rate (HR) is largely under the dual control of the SNS and PNS. The SNS influence on HR is mediated by the release of epinephrine and norepinephrine and activation of β-adrenergic receptors [70]. PNS influences on HR are mediated by the release of acetylcholine from the vagus nerve and muscarinic acetylcholine receptor response increasing K + conductance [71]. Increased SNS or diminished PNS activity results in cardio-acceleration, whereas decreased SNS or increased PNS activity causes cardio-deceleration [72]. Vagal and sympathetic activity constantly interacts, so that under resting conditions, PNS prevails over SNS activity. Dysregulation of the ANS control of the cardiovascular system is associated with increased SNS and reduced PNS tone, a derailment observed both during aging and in patients affected by the most common age-related diseases. The measurement of HR is the simplest biomarker to measure PNS activity [73]. However, the recording of cardiac R-R intervals, called heart rate variability (HRV), has been validated as primarily reflecting PNS modulation of cardiac activity through the vagus nerve [74]. There are multiple domains in HRV measurements, including time domain, frequency domain, and non-linear analysis [reviewed 75. Emerging questions that should be answered to provide potential guidance for clinical practice are related to which of HRV parameters have better performances in risk stratification, which metric can estimate ANS functionality, and which markers can help to monitoring the efficacy of ANS modulating strategies [76]. These issues are currently under investigation, and there are still no definitive results and comprehensive answers.

Normal values for ANS measurements are scarce. Guidelines for measurement have been published, suggesting the importance of recording period length, subject age, and sex, on baseline HRV values [77]. Recommendations suggest measuring resting, reactivity, and recovery HRV, the so called 3 Rs. Free software is today available, making possible calculation of HRV from almost any recording source with any available method [78].

Inverse strong correlation was observed between HRV and circulating well-established biomarkers of inflammaging, such as IL-6 and CRP [79]. HRV was proposed as biomarker for improve risk estimation of cardiac mortality and for prediction and prognosis of chronic cerebral small vessel disease and stroke [80]. Notably, HRV was proposed also as biomarkers in some autoimmune diseases, i.e., RA, showing correlations between reduced vagus nerve tone and increased CRP levels and disease activity [81–83].

Overall, measurement of HRV may contribute to monitor the effectiveness of interventions in patients affected by acute or chronic diseases, using novel treatments involving rebalancing of ANS function [84].

Although not yet validated as a tool for monitoring the activity of the inflammatory reflex specifically, the potential of HRV as a biomarker for stimulation of the inflammatory reflex has been discussed [85]. Interestingly, HRV measurements were proposed not only for the detection of autonomic dysfunction, but also as predictors of increased proinflammatory status in the elderly population [3]. The analysis of healthy subjects revealed enhanced SNS activity in the heart, with increased heart rate and reduced HRV analyzed by 24-h ambulatory ECG monitoring. Notably, the increased heart rate and reduced HRV were associated with a subclinical proinflammatory status, characterized by increased levels of PCR and white blood cell count, thus confirming the presence of an autonomic imbalance with a prevalence of sympathetic tone and its correlation with a low-grade subclinical proinflammatory status in the elderly [3].

On the other hand, data analyzing the possible effects of aging on the hemodynamic component of peripheral chemoreflex are scarce. The results suggest that the hypothesis of the “autonomic derangement” as culprit of age-related changes in chemosensitivity may be true for heart rate responses from peripheral chemoreceptors, but not for systolic blood pressure responses to hypoxia. Moreover, respiratory response from both central and peripheral chemoreceptors seems to be rather independent from sympathovagal balance within the cardiovascular system and barosensitivity [86].

An overactive SNS and reduced PNS activity have become established features of several CVDs, including hypertension, ischemic heart disease, and chronic heart failure, but broadly they could be features of cardiac aging. Notably, cardiac aging is associated with vascular aging characterized by impaired endothelium-dependent vasodilation and defective vessel repair capacity, and these features are associated with severe atherosclerosis and microvascular dysfunction [87–89]. Indeed, modern concepts suggest that cardiac aging can be declined as cardiovascular-inflammaging, since inflammaging in vascular and cardiac tissues is associated with the emergence of pathological states such as atherosclerosis and hypertension [90–92].

## Gender differences in inflammaging and age-related ANS imbalance

Gender differences both in inflammaging and ANS were observed and explained, almost in part, by genetic and epigenetic gender differences and by the effects of male/female sex hormones [93, 94]. Such prevailing hormone levels produce differences not only between men and women, but also between pre- and post-menopausal women [95]. Age at natural menopause is considered a marker of biological aging and it is increasingly recognized as risk factor for chronic diseases and severe outcomes later in life [95]. The aging process shows substantial individual variability between men and women. Life expectancy in women is higher than in men, but women are frailer and have worse health at the end of life, while men still perform better in physical function examinations [96]. Moreover, many age-related diseases show sex-specific patterns, and gender differences were observed in the prevalence of multimorbidity and coexistence of several chronic diseases [97]. Gender differences in key mechanisms that foster inflammaging were highlighted, suggesting that in general women are characterized by lower inflammaging levels during reproductive period [reviewed in 36]. Long-standing evidence support sex-related differences in the activation of the innate immune system and recently gender differences were observed also in “cellular senescence” [98].

Women tend to show a more robust immune response to infection, in part due to the immune-suppressive effects of testosterone and immune-enhancing effects of estrogens [99]. Notably, increased inflammaging levels negatively affect the efficacy of immune responses to pathogens.

Overall, the evidence on gender differences in inflammaging suggests that women may maintain lower inflammaging levels than men during reproductive period, associated with more robust immune responses and reduced risk of diseases than man [100, 101]. This advantage can be exploited in terms of greater survival rate. However, later in life, woman is frailer and in worse health status than man. Old men are expected to be more selected than women.

Gender differences in PNS functions were highlighted primarily in HPA axis. Although the acute HPA response to stressors is a beneficial response, constant activation of this circuit by chronic or traumatic stressful episodes may lead to a dysregulation of the HPA axis [102]. Compared to males, female animal models showed a more robust HPA axis response, because of circulating estradiol levels which elevate stress hormone levels during non-threatening situations and during and after stressors. Fluctuating levels of gonadal steroids in females across the estrous cycle was highlighted as major factor contributing to sex differences in the robustness of HPA activity in females compared to males [102]. Overall, by influencing the response and sensitivity to releasing factors, gonadal steroids help to orchestrate the modulation of the HPA axis.

There is few evidence so far regarding gender differences in the human CAP.

Both animal and human studies have reported sex differences in autonomic control, suggesting that females show greater PNS modulation of cardiovascular activity compared to males [103]. In general, women exhibit higher vagal control of HR potentially due to differences in estrogen and oxytocin, which can enhance efferent vagal activity [104]. Notably, gender difference was observed in the relationship between vagally mediated HRV and CRP levels, supporting the growing body of evidence indicating that females have greater vagally mediated autonomic control than males [105].

Overall, gender difference observed in inflammaging and PNS activity, especially in the activation of HPA axis, reinforce the hypothesis of a crosstalk between the two phenomena.

## Strategies to reduce ANS imbalance in the framework to restrain inflammaging

Targeted multi-organ neuromodulation strategies may beneficially influence multiple aspects of age-related diseases, including cardiometabolic disease [106] and resistant hypertension [107, 108]. Data from sham-controlled clinical trials demonstrate the feasibility, safety, and efficacy of catheter-based renal denervation in subjects with uncontrolled hypertension [109]. In analogy, denervation of the common hepatic artery is now feasible in humans and may prove to be similarly useful in modulating sympathetic overdrive directed toward the liver, pancreas, and duodenum [110]. Notably, some researchers highlighted the underestimation of advanced age as potential factor contributing to poorer response to neuromodulation for cardiovascular diseases [111].

Since we focused our discussion on the relationships between ANS imbalance and inflammaging, highlighting some roles played by CAP and HPA, innovative therapeutic strategies based on modulation of these pathways will be briefly discussed.

CAP activation was proposed for treatment of different age-related diseases. Significant drug discovery efforts have been devoted to identifying several ligands for α7nAChR, with high selectivity and minimal or no side effects to avoid receptor desensitization. Interesting, some of these molecules have shown a therapeutic relevance for the treatment of different neurodegenerative pathologies such as Alzheimer’s and Parkinson’s disease [112]. Considering the effects of α7nAChR activation in astrocytes and microglia in restraining inflammation, the clinical therapeutic potential that α7nAChR agonists may play in the modulation of the neuroinflammation is relevant. However, considering the large distributions of these receptors inside and outside the CNS, the use of these pharmacological ligands could present some limitations. The research for new α7 nAChR selective agonists is still ongoing, trying to reduce or minimize the associated side effects.

Interestingly, the increasing knowledge on the mechanisms of CAP has provided support for clinical trials to evaluate the efficacy of VNS stimulation to treat inflammatory diseases characterized by chronic inflammation, e.g., RA [113]. The anti-TNF-alpha effect of the CAP could be used also in the treatment of chronic inflammatory bowel diseases, such as Crohn’s disease and ulcerative colitis, in which TNF-α plays a key role [114].

Innovative bioelectronic methods of CAP harnessing for clinical use were also assessed [115]. Bioelectronic medicine, via vagus nerve stimulation (VNS), may have an interest in the non-pharmacological therapeutic approach [116]. A recent double-blind, randomized clinical trial examined the effect of chronic low-level transcutaneous VNS on cardiac function in patients with heart failure demonstrating that VNS can have the potential to counteract cardiovascular-inflammaging [117].

Regarding the possibility of intervention on HPA axis, long-lasting changes in the reactivity of the HPA axis to stress were observed during aging and in patients affected by age-related diseases. Several factors seem to be highly relevant in determining the outcomes of such changes, including the nature, timing, and duration of the stressor in combination with the genetic background of the individual and the context in which it is assessed [118]. Although there is a long history of research behind the HPA axis, much remains to be revealed especially in elderly subjects and in patients affected by the most common age-related diseases. It is reasonable to suggest a strong association between the HPA axis responsiveness and the development of the age-related diseases, emphasizing the importance of future studies addressing this gap in our current knowledge.

Overall, the potential benefit for innovative interventions in systemic age-related diseases based on VNS is an emerging field of interest that is worth further study to confirm its efficacy in improving the symptoms and quality of life of the patients.

The recent results encourage the efforts for extending in the future the application of vagal stimulation to healthy elderly subjects in the initial phase of autonomic imbalance that could be monitored through HRV. Future research on this field should be encouraged.

## Conclusion

The progressive age-related autonomic imbalance, characterized by increased SNS activity and decreased PNS activity, contributes to neural- and proinflammatory-mediators outflows, thus fueling inflammaging and increasing the risk to develop the most common age-related diseases, primarily CVDs. The increased systemic levels of proinflammatory cytokines should stimulate the HPA axis response exerting cortisol-mediated downregulation of the proinflammatory status, as well as should activate CAP, inhibiting macrophages and other cytokines producing cells in different tissues (spleen, intestine). During aging, HPA and CAP responses seem to lose efficacy, so that they cannot exert effective anti-inflammaging effects. Gender differences were observed in such phenomena. Macrophages seem to be the cells at the crossroad between reduced PNS anti-inflammaging activity and increased inflammaging levels during aging. Studies on macrophage functions and released biomarkers should be encouraged in this framework. Indices of vagally mediated HRV could be useful biomarkers to estimate PNS activity and inflammaging levels, thus becoming a potential noninvasive biomarker to monitor the trajectories of aging. Consequently, vagal stimulation could be viewed as an attractive therapeutic target to inhibit or attenuate the release of cytokines that promote and perpetuate inflammaging.
